# Fibroblast Growth Factor 8 Expression in GT1-7 GnRH-Secreting Neurons Is Androgen-Independent, but Can Be Upregulated by the Inhibition of DNA Methyltransferases

**DOI:** 10.3389/fcell.2016.00034

**Published:** 2016-05-03

**Authors:** Megan L. Linscott, Wilson C. J. Chung

**Affiliations:** ^1^Department of Biological Sciences, Kent State UniversityKent, OH, USA; ^2^School of Biomedical Sciences, Kent State UniversityKent, OH, USA

**Keywords:** *Fgf8*, GnRH neurons, androgen receptor (AR), olfactory placode, DNA methyltransferases, 5-azacytidine

## Abstract

Fibroblast growth factor 8 (FGF8) is a potent morphogen that regulates the embryonic development of hypothalamic neuroendocrine cells. Indeed, using *Fgf8* hypomorphic mice, we showed that reduced *Fgf8* mRNA expression completely eliminated the presence of gonadotropin-releasing hormone (GnRH) neurons. These findings suggest that FGF8 signaling is required during the embryonic development of mouse GnRH neurons. Additionally, *in situ* hybridization studies showed that the embryonic primordial birth place of GnRH neurons, the olfactory placode, is highly enriched for *Fgf8* mRNA expression. Taken together these data underscore the importance of FGF8 signaling for GnRH emergence. However, an important question remains unanswered: How is *Fgf8* gene expression regulated in the developing embryonic mouse brain? One major candidate is the androgen receptor (AR), which has been shown to upregulate *Fgf8* mRNA in 60–70% of newly diagnosed prostate cancers. Therefore, we hypothesized that ARs may be involved in the regulation of *Fgf8* transcription in the developing mouse brain. To test this hypothesis, we used chromatin-immunoprecipitation (ChIP) assays to elucidate whether ARs interact with the 5′UTR region upstream of the translational start site of the *Fgf8* gene in immortalized mouse GnRH neurons (GT1-7) and nasal explants. Our data showed that while AR interacts with the *Fgf8* promoter region, this interaction was androgen-independent, and that androgen treatment did not affect *Fgf8* mRNA levels, indicating that androgen signaling does not induce *Fgf8* transcription. In contrast, inhibition of DNA methyltransferases (DNMT) significantly upregulated *Fgf8* mRNA levels indicating that *Fgf8* transcriptional activity may be dependent on DNA methylation status.

## Introduction

Traditionally, fibroblast growth factor 8 (FGF8) has been studied in the context of a potent morphogen that is required for establishing morphogenetic centers in the developing mammalian neural tube (Crossley and Martin, 1995; Meyers et al., 1998; Sun et al., 1999). Indeed, *Fgf8* mRNA and protein is highly expressed in the early embryonic midbrain-hindbrain border, and the anterior neural ridge (Crossley and Martin, 1995; Kawauchi et al., 2004). Recently, we showed that FGF8 function is also important for the development of gonadotropin-releasing hormone (GnRH)-secreting neurons, which reside within the preoptic-hypothalamus region. Specifically, while GnRH neurons were present in newborn wildtype *Fgf8* hypomorphic mice, they were absent in homozygous litter mates (Chung et al., 2008). Additional studies showed that GnRH neurons were already missing in the E11.5 *Fgf8* hypomorphic olfactory placode (OP) (Wray et al., 1989; Chung et al., 2008) from which the majority of GnRH neurons emerge (Schwanzel-Fukuda and Pfaff, 1989; Wray et al., 1989). Together these data clearly support the supposition that FGF8 function is required for vertebrate GnRH neuron development (Chung et al., 2008; Tsai et al., 2011).

*In vitro* explant studies in chicken OP explants further pinpointed that FGF8 function is required for the emergence of GnRH neurons. Normally, GnRH precursor cells in the chicken OP are specified around the Hamburger and Hamilton (HH) stage 16/17. Treatment of HH15 OP with FGF8 advanced the emergence of GnRH neurons by ~24 h (Sabado et al., 2012), which was abrogated by a FGF antagonist in HH17 OP (Sabado et al., 2012). These studies not only support the general supposition that FGF8 function is required for fate-specifying GnRH precursor cells, but also that FGF8 only has a narrow window of opportunity to induce the emergence of GnRH neurons.

Although, much is already known about the effects of FGF8 function on GnRH neuron development, it is unclear how *Fgf8* transcription is controlled in the mammalian OP. Data from breast and prostatic cancer cell studies indicate that *Fgf8* transcription is, in part, under the regulatory control of androgen signaling through androgen receptors (AR) (Evans, 1988; Ohuchi et al., 1994; Yamanishi et al., 1995; Gnanapragasam et al., 2002; Tanaka et al., 2002). Specifically, androgen-treatment induced, whereas the androgen antagonist, bicalutamide, inhibited FGF8 protein, and mRNA expression in AR-positive mouse mammary Shionogi carcinoma (SC)-3 cells (Tanaka et al., 1992; Yamanishi et al., 1995). Similarly, androgen signaling increased *Fgf8* mRNA expression in AR positive human prostate LNCaP cells (Yamanishi et al., 1995; Gnanapragasam et al., 2002). These data indicate that androgen signaling may be a general cellular mechanism that induces *Fgf8* expression levels.

In these studies, we tested whether androgen is able to induce *Fgf8* transcription in GnRH neurons. For this purpose, we used the GnRH-secreting GT1-7 immortalized mouse cell line, a model system that has been extensively used in the past to study GnRH neuron biology (Mellon et al., 1990; Wetsel, 1995; Wierman et al., 1995). More importantly, GT1-7 hypothalamic neurons are responsive to androgen signaling because they express classical nuclear ARs (Belsham et al., 1998; Brayman et al., 2012a,b). In addition, we showed that GT1-7 neurons express significant levels of *Fgf8* mRNA, while our previous studies showed that GT1-7 neurons express *Fgfr1* and *Fgfr3* mRNA (Mott et al., 2010). These cellular traits made GT1-7 neurons a suitable model system to study how androgen regulates *Fgf8* transcription at the molecular level in GnRH secreting cells. We first examined two questions: (1) Does AR interact with the 5′UTR promoter region of the mouse *Fgf8* gene in GT1-7 neurons and (2) Does androgen modulate *Fgf8* mRNA levels in GT1-7 neurons and in embryonic mouse OP cells? In addition, because the *Fgf8* gene is enriched for CpG islands, we asked whether changes in DNA methylation affect *Fgf8* mRNA levels in GT1-7 neurons.

Here we will discuss our data demonstrating that while AR interacts with the *Fgf8* promoter region, this interaction was androgen-independent, and that androgen-treatment did not affect *Fgf8* mRNA levels. In contrast, inhibition of DNA methyltransferases (DNMT) significantly upregulated *Fgf8* mRNA levels.

## Materials and methods

### Timed-breeding of mice

Adult 129P2/OlaHsd^*^CD-1 male × female mice were timed-bred in the late afternoon in our animal facility (12L:12D cycle) with access to food and water *ad libitum*. All procedures were approved by the Institutional Animal Care and Use Committee at Kent State University. In the morning, females with a sperm plug were denoted as embryonic day (E) 0.5.

### Androgen response elements in the 5′UTR region of *Fgf8*

**Figure 1:**
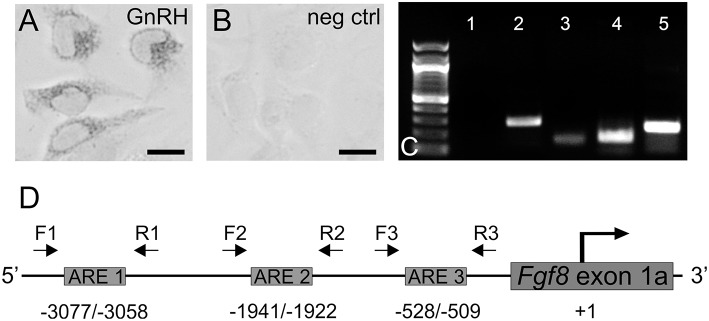
**(A)** Immunocytochemistry of GnRH expressing GT1-7 neurons (gray) and **(B)** negative control (no primary antibody). Scale bar = 25 μm. **(C)** PCR in GT1-7 neurons. lane 1, no cDNA; lane 2, AR; lane 3, *Fgf8*; lane, *FgfR1*; lane 5, *GnRH*. **(D)** Analysis of the mouse *Fgf8* 5′-UTR region [5 Kb upstream of the translation start site (+1)] with Matinspector (Genomatix) revealed the presence of three ARE sites upstream of the translation start site with a shared consensus sequence. Relative position of F (forward) and R (reverse) flanking primer pairs (1, 2, 3) used to detect ARE 1 (−3077/−3058), ARE 2 (−1941/−1922), and ARE 3 (−528/−509).

Identification and prediction of transcription factor consensus sites were determined through MatInspector (Genomatix Software GmbH, Munich, Germany), which indicated the presence of three androgen response element (ARE) sites with a matrix similarity of greater than 0.88 (1 indicating a perfect match) within the 5000 bp 5′ promoter region upstream from the translational start site (Figure 1). The 5000 bp 5′UTR of *Fgf8* was selected due to it's close proximity to the first coding exon in the *Fgf8* gene and the presence of a large CpG island, which is well correlated to promoter regions (Calo and Wysocka, 2013). Given that the 5′UTR has no TATA box, the transcriptional start site(s) are currently unknown (Gemel et al., 1999). Previous literature has also indicated that the human *Fgf8* 5′UTR promoter region lies within this 5 Kb stretch (Gnanapragasam et al., 2002).

### GT1-7 neurons

Immortalized mouse GnRH neurons (GT1-7) (Generously donated by Dr. Pamela Mellon, University of San Diego, CA) were grown in phenol-red free DMEM containing 4.5 g/L pyruvate and 548 mg/L-glutamine, 10% fetal bovine serum (ATCC, Manassas, Virginia), 1% pen/strep (Gemini Bio–Products, Sacremento, California), and 25 μg/ml Plasmocin (InvivoGen, San Diego, California; Mellon et al., 1990). Cells were kept in an humidified incubator at 37°C with 5% CO_2_. For our experiments, GT1-7 neurons were grown to 70–80% confluency, washed three times in a phosphate buffer solution, and cultured for 16 h in DMEM containing 10% dextran-charcoal stripped fetal bovine serum prior to pharmacological treatments (see below).

### Immunohistochemistry in GT1-7 neurons

GT1-7 neurons were fixed with fresh ice-cold 4% paraformaldehyde for 30 min. Following, cells were washed in TBS (3 × 5 min) on a 2D rotator, incubated in primary rabbit polyclonal anti-GnRH (1:5000, generously donated by Dr. Pei-San Tsai, University of Colorado Boulder, CO) made in TBS/0.3% Triton-X (Fisher Scientific, Pittsburgh, PA) and 2% normal goat serum for 2 days at 4°C. Cells were washed with TBS and incubated with biotinylated-goat anti-rabbit (1:600) for 2 h at room temperature followed by ABC (1:800) (Vector Laboratories, Burlingame, CA) in TBS for 2 h at room temperature, and reacted with 0.05% diaminobenzidine (Sigma-Aldrich, St. Louis, MO) containing 0.01% H_2_O_2_ in TBS for 20 min.

### AR chromatin immunoprecipitation in GT1-7 neurons

Chromatin immunoprecipitation (ChIP) was used to examine whether AR interacted with the 5′UTR promoter region of the mouse *Fgf8* gene in GT1-7 neurons in the presence or absence of dihydrotestosterone (DHT). GT1-7 cells were treated with vehicle (0.005% ethanol) or 100 nM DHT for 4 h. Following, GT1-7 neurons were processed for EZ-Magna-ChIP assays (Millipore, Billerica, MA, USA) according to manufacturer's instructions. Briefly, cells were cross-linked with 1% formalin for 15 min and lysed. The protein cross-linked genomic DNA was fragmented to 200–600 base pairs through sonication. Following, the fragments were immunoprecipitated using 1 μg of either rabbit polyclonal antibody against AR (Millipore, Billerica, MA, USA) or control IgG (Millipore, Billerica, MA, USA) for 2 h at 4°C, which was pulled down using agarose-A beads coupled to magnets. Proteinase K (10 mg/ml) was used to reverse crosslinking, and DNA was isolated. The relative amount of AR occupancy on the identified ARE sites in the 5′UTR region of *Fgf8* was measured using using a Mastercycler EP Realplex^2^ (Eppendorf, Hauppauge, NY) with SYBR Green PCR Master Mix (Roche, Basel, Switzerland). For this purpose, three primer sets were designed to flank the identified ARE 1, ARE 2, and ARE 3 sequences (Table 1). ChIP signal was normalized to background by using a 1% input, adjusted to 100%. Non-specific primers flanking upstream of the 5′UTR region of *Fgf8* were used as negative controls for each pull-down. All results were performed in at least three independent experiments.

**Table 1:** **Primer sequences used for detecting ***Fgf8, Fgfr 1, GnRH*** and ***AR*** mRNA expression, and ARE containing DNA sequences within the ***Fgf8*** promoter region**.

**Gene**	**Forward (5′-3′)**	**Reverse (5′-3′)**	**bp**
*Fgf8*	AGAAGACGGAGACCCCTTCG	TGAATACGCAGTCCTTGCCTT	142
*Fgfrl*	ATGGTTGACCGTTCTGGAAG	TGGCTATGGAAGTCGCTCTT	171
*GnRH*	GGCATTCTACTGCTGACTGTGT	CTACATCTTCTTCTGCCTGGCT	252
*AR*	GGACAGTACCAGGGACCAT	CCAAGTTTCTTCAGCTTACGA	288
ARE 1	CTCCACTGATCGCCCATTGT	GCAAATCTCTGCAACTGCGT	396
ARE 2	AGTCATTCTAAAGGCCCCTCA	GACGGACTCCAGTGTCCAAG	83
ARE 3	ACCCGACCCTAAGCAGATCA	TACCCTTGCCTGTCTCTTCCA	132
Non-Specific *Fgf8*	GTCAGTCTGCGAATATAGCTCAG	CACAGTACCAACAAGTGTCACAG	314

### Quantification of *Fgf8* and Fgfr1 mRNA levels in DHT-treated GT1-7 neurons

GT1-7 cells were treated with vehicle (0.005% ethanol) or 100 nM DHT for 2, 4, or 48 h. This DHT concentration is a saturating amount given that the equilibrium dissociation constant (Kd) of AR is ~2 nM (Wilson and French, 1976), and was used in previous studies investigating androgen signaling in GT1-7 neurons (Belsham et al., 1998). Additionally, transfection of human AR-GFP plasmid into GT1-7 cells in the presence of 100 nM DHT was sufficient to translocate AR into the nucleus (unpublished data). Total cellular RNA was extracted with TriPure (Roche, Indianapolis, IN) according to the manufactures instructions. RNA purity and concentration was measured using the Synergy H2 multi-mode reader with a Take3 Micro-Volume plate adapter (Biotek, VT). ProtoScript® II First Strand cDNA Synthesis Kit (New England Biolabs, MA) was used to reverse transcribe 1 μg of total RNA according to the manufacturers instructions. RT-qPCR was performed in triplicate with gene-specific primers (Table 1) using a Mastercycler EP Realplex^2^ (Eppendorf, Hauppauge, NY) with SYBR Green PCR Master Mix (Roche, Basel, Switzerland). Relative *Fgf8* and *Fgfr1* mRNA expression levels were calculated using the ΔΔ^−2CT^ method (Livak and Schmittgen, 2001). Hypoxanthine phosphoribosyltransferase 1 (*Hprt-1*) was used as a housekeeping gene.

### Quantification *Fgf8* and GnRH mRNA levels in E10.5 nasal explants treated with DHT

Adult pregnant wildtype female 129P2/OlaHsd^*^CD-1 mice were sacrificed at E10.5. The uterine horns were quickly removed form the mice and kept in sterile ice-cold phosphate-buffered saline (Sigma-Aldrich, MO). Following, the nasal region containing the OP was surgically isolated using a dissection macroscope, placed on 0.85 μm Durapore membrane filters (Millipore, Darmstadt, Germany), transferred to cell tissue culture inserts (Greiner Bio-One, Kremsmünster, Austria), and grown using the liquid-air interphase method with in phenol-red free Dulbecco's modified Eagle's medium (DMEM)/F12/glutamax (Thermofisher Scientific, MA) supplemented with B27 (Thermofisher Scientific, MA) and 1% pen/strep/myc (Sigma-Aldrich, MO) media. E10.5 nasal explant tissues were collected, immediately flash frozen and kept at −80°C, and used as 0 days *in vitro* (DIV) samples or grown in culture for 3 DIV with media containing vehicle (0.005% ethanol) or 100 nM DHT. Afterwards, total RNA was isolated from all explants (i.e., 0 and 3 DIV). Relative *Fgf8, Fgfr1, GnRH*, and *AR* mRNA expression levels were measured and calculated as described above.

### Quantification *Fgf8* and GnRH mRNA levels in DNA methyltransferase inhibitor-treated GT1-7 neurons

First, GT1-7 neurons were treated with vehicle (0.005% DMSO) or 1 μM 5-azacitidine (AZA) for 72 h. Our AZA dose and length of treatment was based on previous studies showing that these conditions were able to induce gene expression (Xin et al., 2015; Zhou and Hu, 2015). These studies were followed-up in second experiment where GT1-7 neurons were treated with AZA for 72 h in the presence or absence of 100 nM DHT for 4 h. Total cellular RNA extraction and cDNA synthesis were performed (see above). Relative *Fgf8* and *GnRH* mRNA expression levels were measured and calculated as described above.

### Statistical analysis

Data were analyzed using Student *t*-tests or one-way analysis of variance (ANOVA) with treatment and/or DIV as between subject variables. Holm-Sidak tests were used for *post hoc* analysis. Group n's are given in the figure legends. Differences were considered significant if *p* < 0.05.

## Results

### The mouse *Fgf8* 5′UTR regions harbors 3 are consensus sites

Sequence analysis revealed three ARE sequences from the translation start site (+1): 5′-actaaacatgttGTCCcag-3′ (ARE 1: -3077 bp), 5′-tgctttctctgtGTGCttg-3′ (ARE 2: -1941 bp), and 5′-agctgccgtcctGTCCttc-3' (ARE 3: -528 bp) (Figure 1D).

### GnRH, AR, Fgfr1, and *Fgf8* mRNA expression in GT1-7 neurons

In these parametric studies, we established that our GT1-7 neurons were neurochemically similar to those used in other laboratories (Mellon et al., 1990; Belsham et al., 1998; Mott et al., 2010; Brayman et al., 2012b). First, using ICC, we confirmed the presence of GnRH peptide in these cells (Figures 1A,B) Next, using PCR, we verified that our GT1-7 neurons express significant levels of *GnRH, AR*, and *Fgfr1* and *Fgf8* mRNA (Figure 1C).

### AR interacts with the 5′UTR region of *Fgf8* in GT1-7 neurons

**Figure 2:**
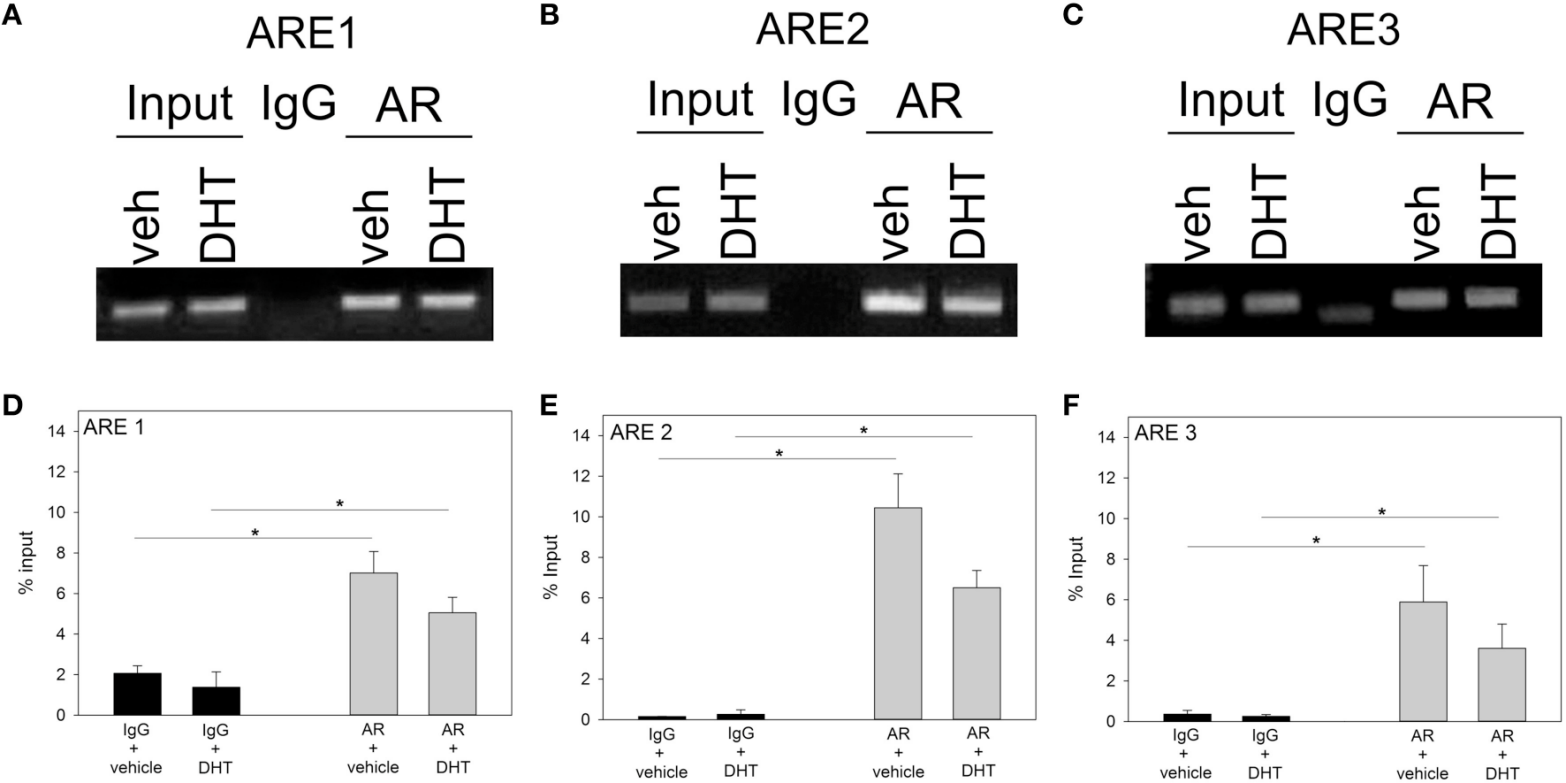
**ChIP gel electrophoresis in vehicle (VEH; ***n*** = 3) or DHT (***n*** = 3) treated GT1-7 neurons (A–C) and qRT-PCR analysis of ARE 1-3 sites (D–F)**. Using an AR-specific antibody, we found that AR interacts with the ARE 1-3 sites as compared to non-specific IgG immunoprecipitation (^*^p < 0.05). However, DHT-treatment did not enhance AR binding to ARE 1–3 sites.

We then asked whether ARs are associated with the 5′UTR region of *Fgf8* in GT1-7 neurons. First, Student *t*-tests showed that ChIP with an AR-specific antibody was able to pull-down more 5′UTR *Fgf8* DNA sequences that contain ARE 1, ARE 2, and ARE 3, in contrast to control IgG (*p* < 0.05; Figures 2A–C). In addition, Student *t*-tests showed that AR interacted with the AREs in a ligand-independent fashion indicating that DHT was unable to affect this interaction (Figures 2D–F).

### DHT did not affect *Fgf8* mRNA levels in GT1-7 neurons

**Figure 3:**
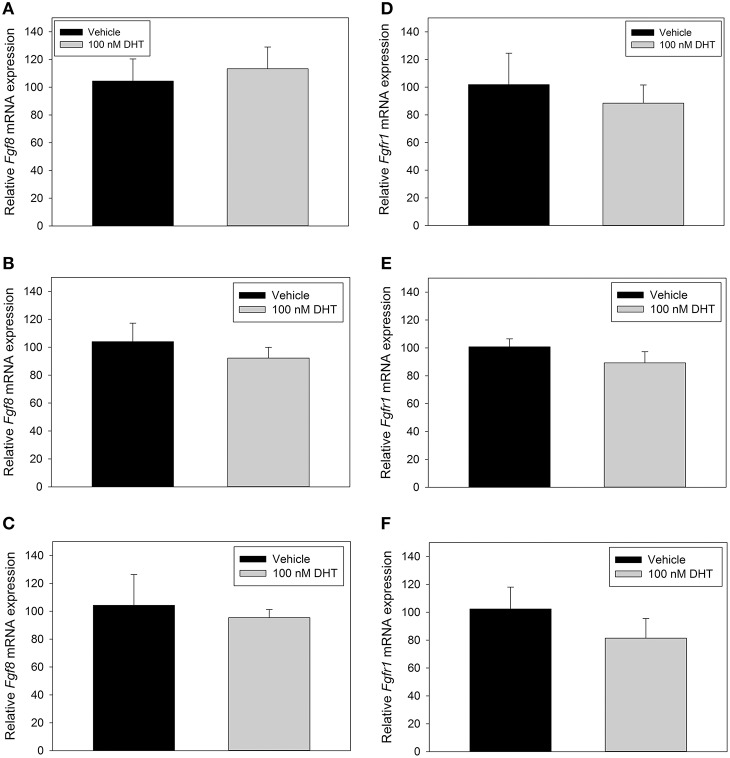
**RT-qPCR for ***Fgf8*** (A–C) and ***Fgfr1*** (D–F) mRNA in GT1-7 neurons treated for 2 h with vehicle (***n*** = 4) or 100 nM DHT (***n*** = 4), 4 h with vehicle (***n*** = 6), or 100 nM DHT (***n*** = 6), or 48 h with vehicle (***n*** = 3) or 100 nM DHT (***n*** = 3)**. DHT treatment did not signifigantly alter *Fgf8* or *Fgfr1* mRNA expression in GT1-7 neurons.

Previous studies showed that androgen was able to induce the mRNA expression levels of *Fgf8* in mouse SC-3 breast and human LNCaP prostate cancer cells (Gnanapragasam et al., 2002; Tanaka et al., 2002). In order to study, whether that was also the case in GnRH-secreting neurons, we treated GT1-7 neurons with 100 nM DHT for 2, 4, or 48 h. Our results showed that neither *Fgf8* (Figures 3A–C) nor *Fgfr1* (Figures 3D–F) mRNA levels were affected by DHT when compared to vehicle-treated GT1-7 neurons.

### DHT did not affect *Fgf8* mRNA levels in nasal explants

**Figure 4:**
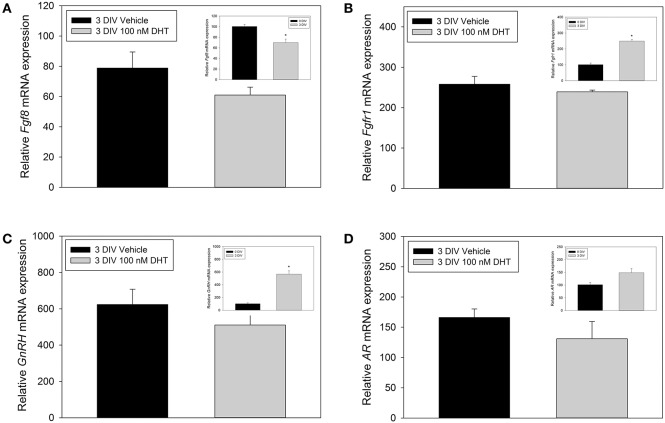
**RT-qPCR for ***Fgf8***, ***Fgfr1, GnRH, AR*** mRNA nasal explants treated for 3 DIV with vehicle (***n*** = 3) or 100 nM DHT (***n*** = 3 animals) (A–D)**. Inset panels represent comparisons between 0 DIV (*n* = 3) vs. collapsed 3 DIV nasal explant expression data (*n* = 6), which showed that *Fgf8* mRNA significantly decreased **(A)**, *Fgfr1* mRNA signifigantly increased **(B)**, *GnRH* mRNA signifigantly increased **(C)**, and no significant difference in *AR* mRNA expression **(D)** after 3 DIV. ^*^p < 0.05.

The inability of DHT to modulate *Fgf8* mRNA expression in GT1-7 neurons may have been due to their immortalized status. Therefore, we used E10.5 nasal explants to investigate whether 3 DIV DHT treatment would be able to increase or decrease *Fgf8, Fgfr1, AR*, or *GnRH* mRNA expression in an organotypic model system. Student *t*-tests showed that *Fgf8* mRNA levels in 3 DIV nasal explants did not differ between vehicle-treated and DHT-treated 3 DIV nasal explant tissue (Figure 4A). Additional analysis showed that *Fgf8* mRNA levels were, however, significantly lower in 3 DIV nasal explants as compared to 0 DIV nasal tissue (*p* < 0.05). Student *t*-tests showed that *Fgfr1* mRNA levels in 3 DIV nasal explants did not differ between vehicle-treated and DHT-treated 3 DIV nasal explants (Figure 4B), although *Fgfr1* mRNA levels were significantly higher in 3 DIV nasal explants as compared to 0 DIV nasal tissue (*p* < 0.05). Student *t*-tests showed that *GnRH* mRNA levels in 3 DIV nasal explants did not differ between vehicle-treated and DHT-treated 3 DIV nasal explants tissue (Figure 4C). As expected, *GnRH* mRNA levels were significantly higher in 3 DIV nasal explants compared to 0 DIV nasal tissue (*p* < 0.001). In contrast, Student *t*-tests showed that *AR* mRNA levels did not differ between vehicle-treated and DHT-treated 3 DIV nasal explants (Figure 4D). On average *AR* mRNA levels seem to be higher 0 DIV nasal tissue compared to 3 DIV nasal explants, but however, did not reach significance.

### Inhibition of DNA methyltransferase upregulated *Fgf8* mRNA levels in Gt1-7 neurons

**Figure 5:**
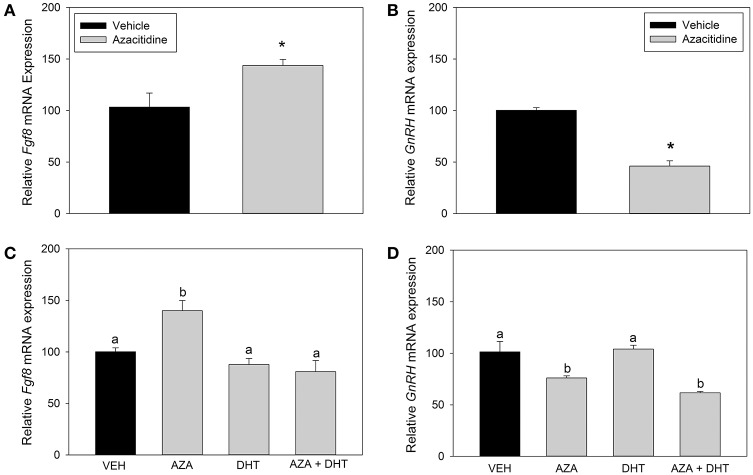
**RT-qPCR for ***Fgf8*** and ***GnRH*** mRNA in GT1-7 neurons treated with vehicle (***n*** = 4) or 1 μM AZA (***n*** = 4) for 72 h**. AZA-treatment significantly increased *Fgf8* mRNA expression (*p* < 0.05) **(A)** and reduced GnRH mRNA levels (*p* < 0.0001) **(B)**. RT-qPCR for *Fgf8* and *GnRH* mRNA expression in GT1-7 neurons treated with vehicle or AZA for 3 days and vehicle or 100 nM DHT for 4 h **(C,D)**. One-way ANOVA showed a significant treatment effect on *Fgf8* mRNA levels [*F*_(3, 15)_ = 10.5, *p* < 0.005] **(C)**. *Post hoc* analysis showed that AZA increased *Fgf8* mRNA levels compared to vehicle (*n* = 4; *p* < 0.01), DHT (*n* = 4; *p* < 0.005) and AZA + DHT (*n* = 4; *p* < 0.005) treatment groups. One-way ANOVA showed a significant treatment effect on *GnRH* mRNA levels [*F*_(3, 15)_ = 13.7, *p* < 0.005] **(D)**. *Post hoc* analysis showed that compared to vehicle and DHT treatment groups, AZA treatment significantly reduced GnRH mRNA levels (*n* = 4; *p* < 0.05). *GnRH* mRNA levels were significantly lower in GT1-7 neurons treated with AZA + DHT (*n* = 4; *p* < 0.005). DHT treatment did not effect GnRH mRNA levels, nor was DHT able to prevent AZA-dependent downregulation of GnRH mRNA levels. ^*^ or different letter indicate *p* < 0.05.

Here, we asked whether changes in DNA methylation affect *Fgf8* mRNA levels in GT1-7 neurons, because the *Fgf8* gene structure is enriched with CpG islands. In our first study, Student *t*-tests showed that compared to vehicle treatment, AZA treatment significantly increased *Fgf8* (*p* < 0.05; Figure 5A) mRNA levels. In contrast, AZA dramatically reduced GnRH mRNA levels (*p* < 0.0001; Figure 5B). In our second study, one-way ANOVA showed a significant treatment effect on *Fgf8* mRNA levels [*F*_(3, 15)_ = 10.5, *p* < 0.005; Figure 5C]. *Post hoc* analysis showed that AZA significantly increased *Fgf8* mRNA levels compared to vehicle (*p* < 0.01), DHT (*p* < 0.005), and AZA + DHT (*p* < 0.005) treatment groups. Interestingly, while we confirmed our earlier results indicating that DHT does not affect *Fgf8* mRNA levels, we found that DHT prevented AZA-dependent increase in *Fgf8* mRNA levels. Moreover, one-way ANOVA revealed a significant treatment effect on *GnRH* mRNA levels [*F*_(3, 15)_ = 13.7, *p* < 0.005; Figure 5D]. *Post hoc* analysis showed that compared to vehicle and DHT treatment groups, AZA treatment significantly reduced GnRH mRNA levels (*p* < 0.05). Similarly, compared to vehicle and DHT treatment groups, GnRH mRNA levels were significantly lower in GT1-7 neurons treated with AZA + DHT (*p* < 0.005). Moreover, compared to vehicle-treatment, DHT treatment did not effect GnRH mRNA levels, nor was DHT able to prevent AZA-dependent downregulation of GnRH mRNA levels.

## Discussion

Several novel discoveries are reported in this study. First, we showed that AR interacts with 3 specific ARE sites (ARE 1: -3077 bp; ARE 2: -1941 bp; ARE 3: -528 bp) in the 5′UTR region of the mouse *Fgf8* gene in GT1-7 neurons. Furthermore, we found that unliganded AR was already recruited to all 3 ARE containing 5′UTR regions, which our data found to be unaffected by the presence of DHT. Second, in contrast to our hypothesis, which was based on previous studies in SC3 and LNCaP cells, DHT did not modulate *Fgf8* mRNA levels in GT1-7 neurons or nasal explants. In contrast, inhibition of DNMT using AZA significantly upregulated *Fgf8* mRNA levels, which concomitant DHT treatment prevented. This suggests that although the 5′UTR region of the *Fgf8* gene allows for AR binding on three separate ARE consensus sites, the function of androgen signaling may not be to upregulate *Fgf8* transcription, but rather to moderate/inhibit the upregulatory effects of other molecular processes, such as DNA methylation.

ChIP assays showed that AR interacts with the *Fgf8* promoter, which suggests that the ARE consensus sites found in the 5′UTR of *Fgf8* are able to enlist ARs in GT1-7 neurons. These results follow earlier studies reporting that ARs were closely associated with the human *Fgf8* promoter region LNCaP cells (Gnanapragasam et al., 2002). However, to our surprise, our DHT treatments did not increase AR recruitment to the mouse *Fgf8* promoter region. Contrarily, earlier findings in LNCaP cells showed that the synthetic non-aromatizable androgen, mibolerone was able to cause enhanced AR recruitment to the human *Fgf8* promoter region (Gnanapragasam et al., 2002). Specifically, these earlier studies showed that mibolerone-dependent AR recruitment occurred only at the most distal ARE consensus site (out of 3 AREs) on the human *Fgf8* promoter region (Gnanapragasam et al., 2002). This discrepancy may be due to technical differences in detection approaches. In our studies, qPCR was used to quantify AR recruitment to the mouse *Fgf8* promoter, whereas AR recruitment to the human *Fgf8* promoter in LNCaP cells was quantified after visualizing the PCR product on film. Alternatively, and more likely explanation for these partially contradictory results may be due to inherent biochemical differences between the androgenic agonists: DHT is an endogenous and naturally occurring androgen, whereas mibolerone is synthetic (Gnanapragasam et al., 2002). Indeed, recent studies showed that although DHT and mibolerone are non-aromatizable androgens, mibolerone may have distinct non-physiological effects on the cell's molecular signaling machinery, such as that mibolerone can activate progestin receptors in breast cancer cells, a non-physiological effect that DHT does not exhibit (Cops et al., 2008). Similarly, the expression of some miRNAs in LNCaP cells, such as miR-120 can be induced in the presence of mibolerone, but not DHT (Segal et al., 2015). Taken together, we hypothesize that AR recruitment to the *Fgf8* promoter under normal physiological conditions is independent of the cellular and extracellular androgen milieu, however a more extensive analysis of AR/ARE function are needed to definitively rule out an androgen-mediated effect on *Fgf8* expression.

The lack of DHT-dependent induction of *Fgf8* mRNA in GT1-7 neurons was unexpected, especially in light the data from earlier studies in SC3 and LNCaP cells (Ohuchi et al., 1994; Yamanishi et al., 1995; Gnanapragasam et al., 2002; Tanaka et al., 2002). The ability of AR to drive transcription depends on the recruitment of specific co-regulators, which may act directly or indirectly with AR to modulate its ability to activate gene transcription (Chung and Auger, 2013). It is well-known that in addition to the presence of its cognate androgenic ligand, the cellular complement of co-factors, such as SRC-1 and ARA-70 plays a role in AR's ability to drive transcription, and that this complement is cell type dependent. Therefore, it is possible that GT1-7 neurons do not have the pre-requisite complement of co-factors that allows DHT to appropriately activate AR to induce *Fgf8* mRNA levels as it is the case in SC3 and LNCaP cells.

Alternatively, regulation of *Fgf8* transcription may be under the control of DNA methylation status (Chung and Auger, 2013), an inference that is supported by our results. In addition to harboring multiple CpG islands upstream and downstream of the translation start site (http://www.ncbi.nlm.nih.gov/epigenomics/), we found that AZA induced *Fgf8* mRNA levels in GT1-7 neurons. Although, we currently do not known whether AZA directly changed the DNA methylation status of the *Fgf8* promoter, our results follow previous studies indicating that hypomethylation in primary rhabdomyosarcoma tumors was correlated with higher *Fgfr1* mRNA expression levels in (Goldstein et al., 2007). These studies confirmed earlier experiments in which DHT treatment for 4 h does not affect *Fgf8* mRNA levels; however, to our surprise, DHT did eliminate the AZA-dependent rise in *Fgf8* mRNA levels. We speculate that in contrast to our original hypothesis, androgen signaling may act to act downregulate DNA methylation-dependent upregulation of *Fgf8* mRNA expression, and hence limit the morphogenetic or proliferative effects of FGF8 signaling (Ornitz and Itoh, 2015). Nonetheless, these results indicate that changes in DNA methylation status can induce *Fgf8* transcription, and that androgen signaling is functional.

In contrast, AZA caused a dramatic decrease in *GnRH* mRNA levels in GT1-7 neurons. This result was unexpected, because evidence from earlier studies in Rhesus monkey nasal explants showed that demethylation of the *GnRH* promoter correlates with a rise in GnRH mRNA levels (Kurian et al., 2010). This discrepancy may be due to the inherent differences between our experimental model systems. Alternatively, and more likely, the decrease in GnRH mRNA levels may be related to the increase in *Fgf8* mRNA expression. Indeed, previous studies showed that treatment with recombinant FGF8b protein not only reduced *GnRH* promoter activity, but also *GnRH* mRNA levels in GT1-7 neurons (Mott et al., 2010). Currently, we are studying whether FGF8 signaling overrode the rise of GnRH mRNA levels due to the DNA demethylating effects of AZA.

In conclusion, our data showed that while AR interacts with the *Fgf8* promoter region, this interaction was androgen-independent, and that androgen treatment alone did not affect *Fgf8* mRNA levels, indicating that androgen signaling does not induce *Fgf8* transcription in GT1-7 GnRH-secreting neurons or nasal explant cells. In contrast, our studies did show that inhibition of DNA methyltransferases significantly upregulated *Fgf8* mRNA levels indicating that *Fgf8* transcriptional activity may be dependent on methylation status.

## Author contributions

ML was responsible for developing the main hypothesis, experimental design and procedures, data analysis and writing of the manuscript. WC contributed to the development of the main hypothesis, experimental design, data analysis, and writing of the manuscript.

### Conflict of interest statement

The authors declare that the research was conducted in the absence of any commercial or financial relationships that could be construed as a potential conflict of interest.
